# Remediation of diesel-contaminated soil enhanced with firefighting foam application

**DOI:** 10.1038/s41598-020-65660-3

**Published:** 2020-06-01

**Authors:** Joanna Rakowska

**Affiliations:** Institute of Safety Engineering, The Main School of Fire Service, Słowackiego 52/54, Warsaw, 01-629 Poland

**Keywords:** Environmental sciences, Engineering

## Abstract

During rescue operations related to the elimination of the effects of industrial accidents or natural disasters, extinguishing agents are used that affect the migration and transformation of contamination in the environment. Firefighting foam sprayed onto an oil spill slowly drains to an aqueous solution and penetrates the soil. The role of surfactants in the removal of petroleum derivatives is well known, but such extinguishing agents also contain solvents, preservatives, corrosion inhibitors and other ingredients that can reduce the beneficial effect of surfactants on soil remediation. The article presents the results of research on the remediation of soil contaminated with diesel fuel and enhanced with firefighting agents used to extinguish fires or remove oil spills on the road. The obtained results of biodegradation and leaching studies indicate differences in the efficiency of diesel fuel removal from soils. It was also found that Wet 1% reduces the amount of polycyclic aromatic hydrocarbons (PAHs) in the soil compared to oily samples not wetted with extinguishing solutions. Chromatographic analyses have shown both the hydrocarbons degradation and the possibility of their transformation into more hazardous compounds. The effectiveness of soil remediation depends on the chemical composition of the extinguishing agent used on the contaminated soil.

## Introduction

Industrial development is related not only with the production of new goods and achieving better standards of living but also with changes in the natural environment. Soil contamination caused by petroleum and its derivatives is one of the most widespread environmental problems^1^. Approximately 0.1–0.25% of crude oil gets into the aquatic and groundwater environment^2^. It also contaminates aquifers and sources that are often used for urban and industrial purposes^3^. Among the petroleum products that are most commonly released into the environment are fuels (their production accounts for 80–83% of processed crude oil)^4^. Oil refineries, storage depots for crude oil and petroleum products, gas stations and industrial plants are exposed to contamination with hydrocarbons. Oil derivatives are also responsible for major contamination of military base areas, as well as the places where incidents have occurred during the transport of crude oil or products derived from its processing^2^. Among the crude oil products released into the ecosystem the largest amount are from fuels used to power vehicles or transported as cargo. A particularly difficult contaminant to deal with is diesel oil as it consists of many compounds of different chemical structures and biodegradability. Diesel oil is characterized as a low evaporation rate liquid with slow degradation rates compared to other petroleum derivatives. Each of diesel oil compounds has a different impact on soil microorganisms and may be present in different concentrations.

Petroleum products are complex mixtures of hydrocarbons, some of which are readily absorbed by organic matter in soil and are very persistent in the environment. All these substances belong to non-polar, lipophilic (hydrophobic) compounds and are very slightly soluble in water. These hydrocarbons are harmful to aquatic and soil organisms, therefore, a release of large amounts of them into the environment may pose a long-lasting risk due to adverse changes in ecosystems and their danger to human health and life. Petrochemicals have strong toxic, carcinogenic and mutagenic properties^5,6^. Hydrocarbons present in the soil can hinder air exchange and reduce water capacity resulting in the filling of soil pores with oil derivates, inhibiting the growth of plants. Residual hydrophobic organic compounds damage the soil environment and finally impact human health as a link in the food-chain and through bioaccumulation^7^.

Contamination by hydrocarbons can be dangerous because some compounds can remain in the environment for a long time. In the case of humans and animals, exposure, to volatile organic compounds may lead to irritation of the respiratory or digestive tract and changes in the nervous system^8,9^. A study on the correlation of polycyclic aromatic hydrocarbon (PAH) concentrations in soils with the effects on human health revealed incremental lifetime cancer risks^10^.

Spills of crude oil or its products ought to be removed from the environment, due to their toxicity and persistently harmful impact on living organisms. In order to successfully achieve environmental restoration, many remediation technologies have been developed and applied^9,11,12^. One of them is bioremediation, in which microorganisms use oil hydrocarbons as a source of energy. The purpose of this process is to transform noxious pollutants adsorbed in the soil matrix into less harmful or non-toxic compounds^13,14^. Microorganisms can convert hydrocarbons to biomass, CO_2_, and H_2_O^15,16^. Although relatively slow, the biological decomposition of hydrocarbons^17^ is an often used technique because of its simplicity, ability to be implemented even over large areas, environmental friendliness, low-cost and high efficiency^18^.

The biodegradation of oil in soil depends on many factors, including both the chemical structure of the hydrocarbons as well the type of soil, the microbial consortium, and environmental conditions, such as the temperature, humidity, pH, presence of nutrients and oxygen^1,9,19^. If conditions which are inhibitory or toxic to microorganisms occur in the soil, the contamination will not be biodegradable even in the presence of organic matter, nitrogen and phosphorus and favorable environmental conditions^20^. Simpler hydrocarbons with lower molecular weight are more readily biodegraded than heavy crudes.

Increasing the effectiveness of the biodegradation of pollutants requires improving their bioavailability for microorganisms. One of the technological factors affecting the biodegradation of petroleum hydrocarbons is the use of surfactants. These substances change the interfacial tension between contaminating particles and soil, and can transfer hydrocarbons to the mobile phase^8,21^. This may cause dispersion of non-aqueous phase liquid (NAPL) droplets in the soil solution, stabilization of emulsions and solubilization of oil into the core of aggregates of surfactant molecules^13,22^.

Industrial accidents may result in fires and explosions which can cause loss of life or injury, property damage and environmental pollution. Events related to the combustion process require the use of water or firefighting foam to extinguish the fire while oil spills are removed by dispersing agents or adsorbents. For this reason possible co-contamination with diesel and firefighting foam or dispersing agent can intensify remediation in the soil or have a persistent harmful impact on an ecosystem^3^. Moreover, these agents mixed with other chemicals or residues of burnt hydrocarbon can also remain in the soil for a long time. These contaminants can be adsorbed by soil particles, released into the air or can contaminate surface and groundwater, which is often used to supply urban or industrial water^23^.

Surfactants are the main component of extinguishing agents like concentrates of firefighting foam. Other components of these foaming agents include organic solvents, foam stabilizers and corrosion inhibitors. Firefighting foams produced through the mechanical dispersion of air bubbles in liquid are used to control and extinguish the combustion of solid materials, flammable liquids and liquefiable solids or gases.

The simultaneous contamination of soil with petroleum hydrocarbons and the extinguishing or dispersing agent can have a significant impact on the remediation process. Preparations used to extinguish fires or remove spills of liquids on the roads contain in their composition surfactants whose impact on soil remediations has been proved^1,8,16,23–25^. However, the interaction of surfactants and other components of these compositions can inhibit the degradation of hydrocarbons by microorganisms.

The general focus of this research was to relate the course of the biodegradation process in soil samples contaminated by diesel oil in the presence of extinguishing agent solutions. This research used the respirometric method, gravimetric and chromatography analyses to evaluate the impact of extinguishing foam and dispersing solution on the remediation of soils contaminated with diesel oil.

## Materials and Methods

### Materials

Diesel oil (grade B) was obtained in an Orlen petrol station (http://www.orlen.pl/). The characteristic parameters of oil are: cetane number – 55, cetane index – 46, density 840 g ∙dm^−3^, viscosity 4 mm^2^/s at 20 °C.

The study was carried out on three types of ground materials which were taken from agricultural land, in the central part of Poland, 10–15 km from the city of Piotrków Trybunalski. The studies were carried out on three types of soil, which in various proportions contain: sand, limestone, clay and peat. The properties of the soils are shown in Table 1. Laboratory contaminated soil samples with 10 ml/kg of diesel oil were prepared and left to stand at room temperature for 24 h until subsequent use.

**Table 1:** Properties of studied soils.

Properties		Soil sample		
		P	R	S
Organic Matter (%)		84.2	38.1	0.2
Mineralogy (main components)		quartz	quartz, limestone	quartz
Grain size (mm)		0.05–4	0.10–10	0.05–2
Texture (%)	sand	9	44	97
	silt	4	16	2
Density (g∙cm^−3^)		0.198	0.926	1.782
Porosity (%)		89	46	23
pH		4.7	7.4	6.7

For test purposes firefighting concentrates and dispersing medium with different chemical compositions and application scope were used. Properties of the studied agents tested at 20 °C are shown in Table 2.

**Table 2:** Properties of firefighting and dispersing agents.

Agent	Main ingredients	Density, g/ml	pH	Surface tension, mN/m	Biodegrability, %
Synt 3%	surfactants (fossil) 10÷20%, organic solvents 15÷30%, corrosion inhibitors <1%, foam stabilizers <2%, water	1.05	7.1	28.0	88
AFFF 3%	surfactants (fossil) 1÷10%, fluoroalkyl surfactants 1÷5%, organic solvents 15÷40%, magnesium sulphate <5%, ethylene glycol <20%, water	1.06	7.5	18.5	76
Wet 1%	surfactants (from fossil and renewable raw materials) 10–40%, aliphatic alcohol <1%, water	1.03	7.6	29.8	98
Det 2%	surfactants (from fossil and renewable raw materials) <20%, organic solvents <40%, aliphatic alcohols 0,1÷1%, water	1.01	7.6	29.5	94

In addition, a biopreparation containing *Bacillus subtilis* and *Bacillus licheniformis* strains (250 ∙ 10^6^ bacterial cells of each type in 1 ml) was used to accelerate the decomposition of hydrocarbon residues.

### Washing procedure

To determine the interaction of water and oil phase in porous structures, studies on leaching diesel from soil were carried out. A washing medium solution of firefighting agents or dispersing agents which are used to remove oil spill from roads were used. Contaminated soil samples each weighing 100 g were placed in glass columns and then washed with the 1000 ml of the studied solution. Samples of the leaked liquid containing the washed oil-water mixture were separated in the separating funnel. The elution efficiency was calculated as the ratio of washed out oil volume to the contaminant oil volume.

It was assumed that the values measured in this way are proportional to the content of the oil phase. The test was repeated 5 times for each soil/washout solution system.

### The rate of oxygen consumption

The rate of biodegradation of hydrocarbons from diesel oil under aerobic conditions was evaluated on the basis of the measurement of the oxygen consumption of the microorganisms contained in the polluted soil. In order to compare biological oxygen demand (BOD) in the contaminated soils, the respirometric method with the OxiTop Control set was used. The study examined the impact of different solutions of firefighting or dispersing agents. In test bottles an examined sample of contaminated soils with nutrients (19.7 g/l K_2_HPO_4_, 7.26 g/l KH_2_PO_4_, 1.8 g/l H_2_NCONH_2_, 0.2 g/l MgSO_4_ · 7 H_2_O) and inoculated with the 2 ml of *Bacillus subtilis* strain (250·10^6^ bacterial cells in 1 ml) and *Bacillus licheniformis* strain (250  · 10^6^ bacterial cells in 1 ml) was closed for a period of 30 days. The released carbon dioxide was absorbed by sodium hydroxide NaOH, a nitrification inhibitor in concentration 20 droplets/l was added. The biological oxygen demand was calculated using Eq. (1).

1 $$BOD=\frac{M({O}_{2})}{R\cdot {T}_{m}}\cdot \left(\frac{{V}_{total}-{V}_{s}}{{V}_{s}}+\alpha \frac{{T}_{m}}{{T}_{o}}\right)\cdot \varDelta p({O}_{2})$$

where: BOD - biological oxygen demand, mg/dm^3^; M (O_2_) - molar mass of oxygen (32 g/mol); R - gas constant (8.3144 J∙mol^−1^⋅K^−1^); T_m_ - measuring temperature (296.15 K); T_0_ - temperature (273.15 K); V_total_ - bottle volume, mL; V_s_ - sample volume, mL; α - Bunsen absorption coefficient (0.03103); ∆p (O_2_) - difference of the partial oxygen pressure, hPa.

In all the experiments, demineralized water with conductivity equal to 6 µS was used.

### Analyses of hydrocarbons

Soil samples with 50% humidity, contaminated with 10 ml/kg of diesel oil, each weighing approx. 500 g, were placed in polyethylene boxes (21 cm × 26 cm × 7 cm) and wetted by spraying with 50 ml of water or firefighting agent solution every 72 h to maintain the soil humidity. Experiments were conducted in two series: 30 days and 60 days, both at 23 °C. After these periods the analysis of the hydrocarbon content in the soil was carried out applying a Soxhlet extraction of a chemically dried soil using 1:1 dichloromethane/acetone followed by gas chromatography mass spectrometry (GC/MS) instrumental analysis according to the appropriate method described in standards^26–28^. All samples were analyzed using an Agilent 7890 A GC (gas chromatograph) equipped with a 5975 C MS (mass spectrometer).

## Results and Discussion

### Removal of diesel

The washout of petroleum derivates from the soil due to the action of extinguishing agents is associated not only with the type of substance used, but also with the chemical properties and physical structure of the substrate. The diesel fuel was removed most effectively from all of the tested types of soil with a 2% solution of agent Det, which washed out from 30 up to 75% of the oil from the tested soils. This may be due to the use of a composition containing surfactants which are wetting, emulsifying and solubilizing the water-repellent contaminants. In consequence, the particles were removed from the soil grains. The effectiveness of oil removal obtained in this study was found to be in accordance with that in the existing literature. In the study^29^, oil washout using a solution of nonionic surfactant Tween-20 was 18%, while the use of system surfactant/ethylene glycol increased the efficiency of diesel removal up to 62%^30^. It was found that for anionic surfactant, additional amounts of ethylene glycol may reduce the oil removal effect. They found that for anionic surfactant, increasing the addition of ethylene glycol may reduce the oil removal affect. Therefore, the amount of ethylene glycol in AFFF 3% is important for leaching efficiency. Additionally, AFFF 3% despite having a greater ability to reduce the surface tension of water, has rather poor wetting and emulsifying properties, which does not allow effective removal of hydrophobic contaminants such as diesel. The highest cleaning efficiency was observed from the substrate P with a high content of peat. This effect can be explained by a reduction in the interfacial tension between the diesel/soil caused by the detergent solution and a decrease of the wetting angle of the hydrophobic surface. This results in a reduction in the adhesion of the contamination to the soil, solubilization of lipophilic substances in the core of micelles and an increase in their mobility in the very narrow spaces between the soil skeleton. Soil structure is also important, i.e. the size of the particles.

**Figure 1:**
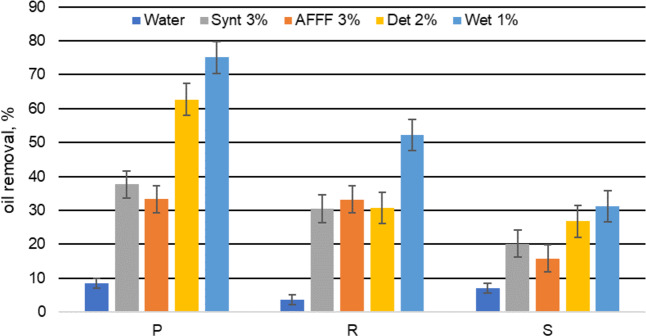
The efficiency of the leaching of diesel oil from soils by studied solutions.

The tests were repeated 7 times for each soil/solution system and the average standard deviation was equal to 4.85%. The Pearson correlation coefficient (*r* = 0.886) showed that the oil removal from the soil was significantly positively correlated with the type of washing solution. Statistical significance value (*p*) was equal 0.046. The results of diesel oil removal from the studied grounds are illustrated in Fig. 1.

### Biodegradation

Respirometric biodegradation tests were conducted on the peaty, rendzina and sandy soil samples polluted with diesel oil. The demand for oxygen in raw samples and in systems containing foam solutions was compared. The highest rate of oxygen consumption was observed in the samples with the addition of the Wet 1% and Det 2% solutions. The least efficient process was found in a sample with the AFFF 3% solution containing fluorosurfactants, which are more difficult to biodegrade and are long lasting in the environment.

**Figure 2:**
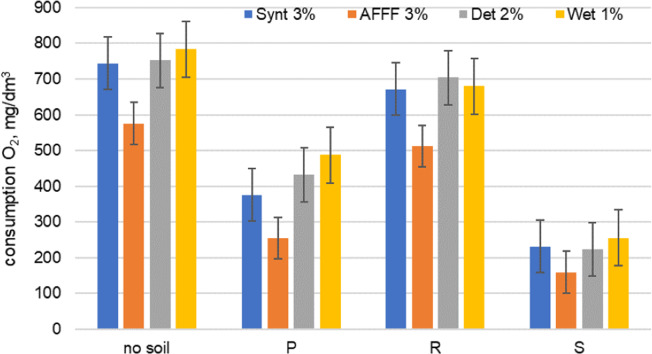
Biodegradation of diesel oil with solution of firefighting agents or dispersant.

The obtained results confirmed that in the presence of an extinguishing medium which is less susceptible to biodegradation the remediation process was less effective than in the case of the use of the firefighting composition containing substances not toxic to microorganisms and readily biodegradable. Surfactant toxicity is an important factor which may adversely affect the biodegradation of oil and petroleum hydrocarbons. A high concentration of surfactants can inhibit microbial growth in soil which is rich in organic matter^3,24^. The remediation process of the soil substrate with the addition of agents Det 2% and Wet 1% was clearly better than with AFFF 3% (Fig. 2). In the AFFF 3% solution there are fluorosurfactants and solvents which may be toxic for microorganisms and inhibit their biological activity. The process of biodegradation of the analyzed pollutants proceeded faster in the soil with lower amounts of organic matter (type R) than in the peaty soil (type P). This can be explained by the high content of organic matter in P substrate, which is a more preferable source of energy for microorganisms than petroleum compounds and surfactants. In addition, it should be remembered that hydrocarbon substances with a similar structure to petroleum compounds may naturally occur in soil and also degrade, transform and bioaccumulate^31^. The difference in the oil biodegradation in soil is also associated with the process of sorption of pollutants and their immobilization. The biodegradation of hydrocarbons sorbed on soil particles is less efficient than the degradation of hydrocarbons in the liquid and NAPL phases^32^. Therefore, greater liquid retention results in higher oxygen consumption to diesel biodegradation in soil (type P and R). All tests were repeated 5 times. The biodegradation of diesel show strong correlations with the type of extinguishing or dispersing agent used to remediation of soil (*r* = 0.947, *p* = 0,035). The test results are shown in Fig. 2.

### Content of petroleum derivates

In order to assess the progress of the soil remediation process, pollution concentrations were analysed at 30 and 60 days after contamination. Qualitative and quantitative examinations of the hydrocarbons in the soils were made according to standard procedures. The level of hydrocarbon detection in the soil with these methods was below 0.010 mg/kg. For the results of each of the analysed substances, an extended uncertainty was calculated for the confidence level equal to 95%^26–28^.

A comparison of the hydrocarbon content in soil immediately after contamination (10 ml/kg) and then after 30 and 60 days allows us to conclude that as a result of the remediation process, the amount of oil derivatives was significantly reduced. The main mechanism of soil cleaning was the evaporation of the hydrocarbons. Both at 30 and 60 days after the contamination, about a 3 times higher concentration of mineral oil was observed in soil with a higher content of organic matter (soil P). Such results may also be related to the physical parameters of the ground, for example the porosity, as well as the presence of other sources of carbon (organic matter content) preferred by microorganisms. Petroleum hydrocarbons in soil are not the sole source of carbon and energy, and their biodegradation process does not always result in a complete degradation (mineralization) of organic pollutants. In fact, the microorganisms transform the substances through metabolic or enzymatic reactions which are based on two processes: growth and cometabolism. In growth, an organic pollutant is used as the sole source of carbon and energy. The other process of degradation – cometabolism, is defined as the metabolism of an organic compound in the presence of a growth substrate that is used as the primary carbon and energy source. Besides the above mentioned biodegradation processes when a pollutant compound is used as a carbon source or a compound is enzymatically attacked but is not used as a carbon source (cometabolism) bioremediation researchers distinguish one more path of removal from the soil matrix - contamination is taken up and concentrated within organisms (bioaccumulation)^33^.

**Figure 3:**
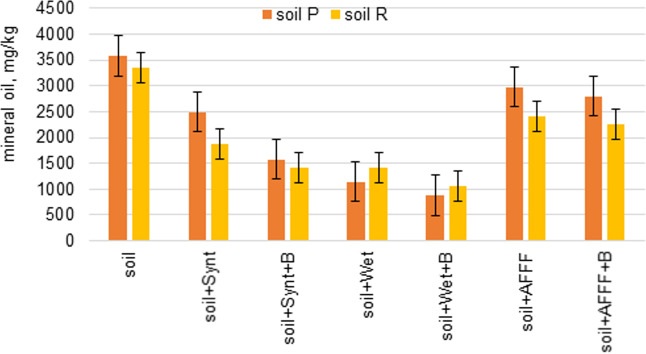
Content of mineral oil in soil 30 days after contamination (B – bacteria strains).

The presence of substances containing surfactants in the contaminated soils resulted in slightly lower (about 5%) diesel oil residues in the tested samples. The lowest hydrocarbon content was observed in samples with Wet 1%. This firefighting agent contains surfactants obtained from renewable raw materials. Karthik’s research^34^ also showed that alkyl polyglucoside (biosurfactant) allows 10% to 50% oil removal. The amounts of the mineral oil content 30 days after contamination in different types of soil are shown in Fig. 3.

On the basis of a study of the hydrocarbon content in soil P and R contaminated by diesel, after 30 days a significant loss of contamination by evaporation was observed. Porous materials with bigger particles blocked more diesel oil volatilization in comparison to finer sized particles. This proves that diesel oil volatilization depends upon the mean grain size of the porous media^35^. The impact of soil structure and its composition on the efficiency of diesel leaching was also observed by other researchers^34^.

A higher mineral oil content was detected in soil P than in soil R. This is probably due to the different structure of these soils and their ability to retain liquids in the porous structure. The introduction of bacteria strains in contaminated soils promotes the decomposition of mineral oil. The average results of the mineral oil concentration in tested samples are shown in Fig. 3. The correlation coefficient (*r*) showed a strong relationship between the mineral oil content and the presence of microorganisms and firefighting agents across the seven soil systems (*r* = 0.952, *p* = 0.001).

In order to assess the progress of the soil remediation process, pollution concentrations were analysed at 30 and 60 days after contamination. Quantitative indications of the hydrocarbons in the soil were made according to standard procedures. The level of hydrocarbon detection in the soil using these methods was 0.010 mg/kg^26–28^.

The results of the hydrocarbon concentrations in the studied soil show that the amount of contamination in R sample is lower than in the S and P samples. Comparing the hydrocarbon content in soil 30 or 60 days after contamination, faster soil remediation was found in the presence of extinguishing agent or dispersant solutions. Moreover, various rates of individual PAHs degradation in different soils have been observed. Other researchers also observed a similar relationship^10,36^. Perhaps the proportions between the quantities of analyzed hydrocarbons in the studied soils were different due to the presence of various types and amounts of native organic matter and nutrients.

**Figure 4:**
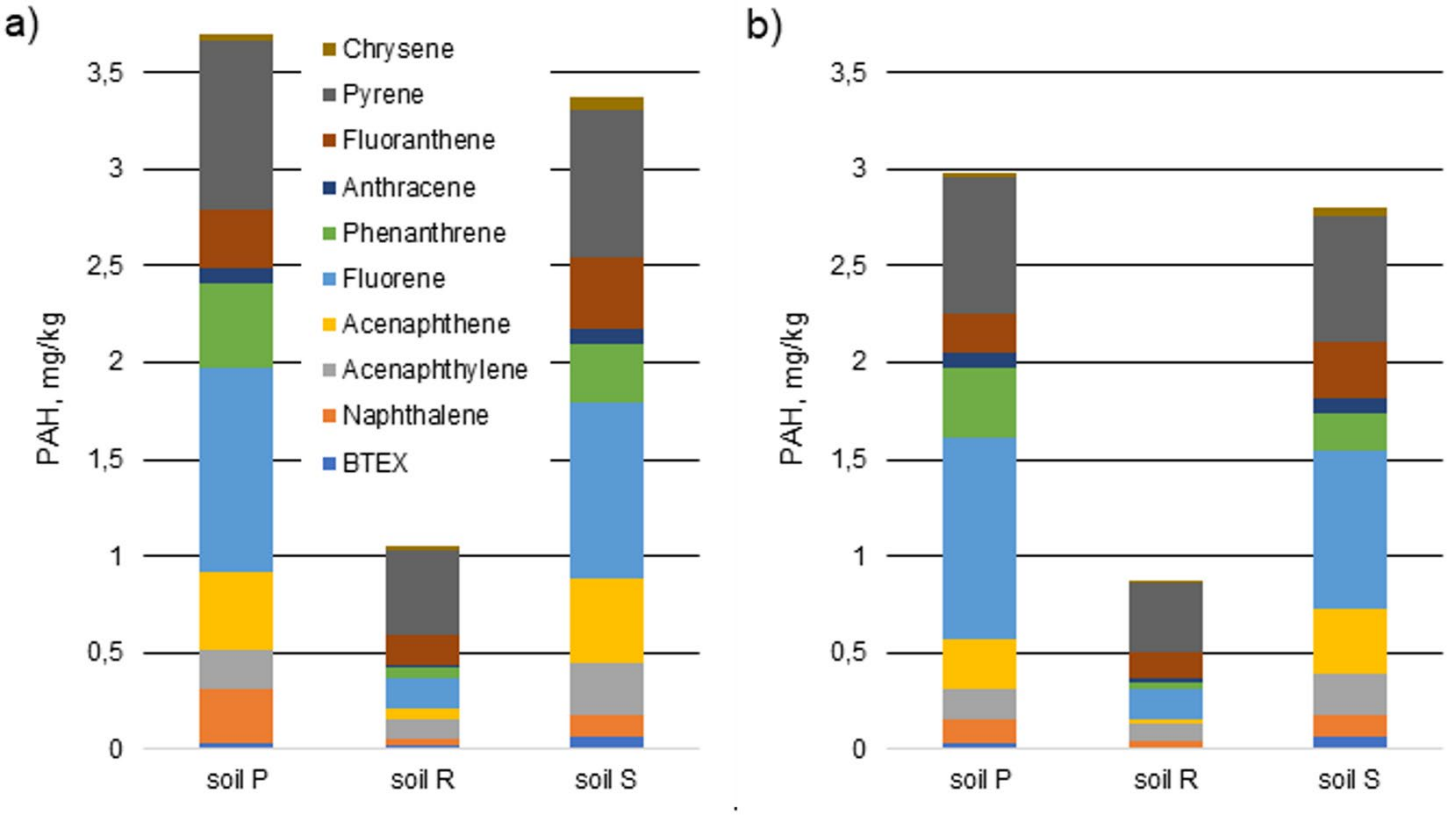
Content of hydrocarbons in contaminated soils after 30 days of remediation: (**a**) no firefighting agent, (**b**) with firefighting agent Synt 3% (*r* = 0.987, *p* = <0.001).

**Figure 5:**
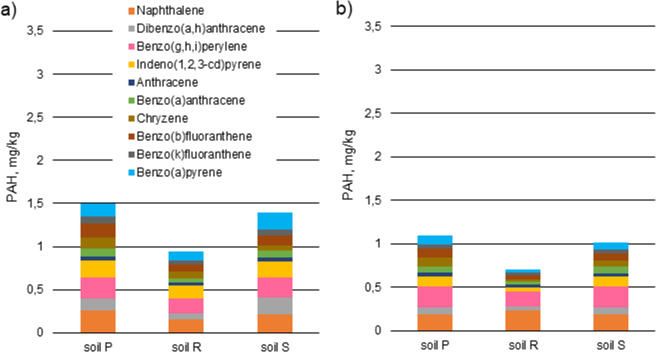
Content of hydrocarbons in contaminated soils after 60 days of remediation: (**a**) no firefighting agent, (**b**) with firefighting agent Synt 3% (*r* = 0.984, *p* = <0.001).

All 16 PAHs were detected in soil samples but their composition pattern in the 30 day and 60 day samples were different (Figs. 4 and 5). The PAHs with 2–4 rings comprised the majority of PAHs in soil samples examined 30 days after contamination while in samples tested 60 days after pollution PAHs with 4–6 rings predominated. This could be due to the faster biodegradation rate of PAHs with lower mass by soil bacteria. As Ma^34^ noted, the heavy components of diesel oil probably could be closed in soil capillaries and adsorbed on the surface of the skeleton. However, the content of similar amounts of naphthalene in 30- and 60-day samples may be caused by a strong association with soil organic matter, thus reducing its losses by volatilization^37^.

The presence of some compounds, e.g. the most carcinogenic of all PAHs, was found only in samples after 60 days of decomposition. This means that hydrocarbons undergo various transformations that can result in the formation of more hazardous substances than the primary pollutants. The results of testing the content of the detected hydrocarbons occurring in the soils are shown in Figs. 4 and 5. The Pearson correlation coefficients (*r*) and statistical significance (*p*) values are given below the charts.

Across the world, different permissible levels of soil pollution with polycyclic aromatic hydrocarbons are applicable. In this paper the Dutch Target Value of Soil Quality for PAHs in soil^38^ was used to compare with the concentration of individual PAHs present in the tested soil.

- Soil quality criterion - 1.5 mg of PAH/kg of soil. The criterion indicates a safe level for contact with soil (children playing, home owners).
- Cut-off criterion - 15 mg of PAH/kg of soil. This criterion indicates the level at which all contact with soil should be avoided if the land use of the area is sensitive.

The concentrations of PAHs in R soil 30 days after contamination were safe for people in contact, while 60 days after pollution in all tested samples polycyclic aromatic hydrocarbons did not pose a risk for children and adults.

## Conclusions

During rescue operations that are a response to industrial accidents or natural disasters, extinguishing agents are used that affect the migration and transformation of pollution in the environment. Their role in these processes is primarily associated with the impact of surfactants and solvents on the behavior of petroleum derivatives in the ground. The effectiveness of soil remediation depends on many factors, including the chemical composition of the firefighting agent or dispersant applied to the contaminated ground. The properties of the preparation solutions: the ability to lowering of surface tension, capacity for emulsification and micellar solubilization of hydrocarbons, and their toxicity to microorganisms are the main parameters affecting the petroleum derivates’ degradation in soil in the presence of firefighting agents or dispersant. This study proved the impact of the type of soil on their remediation when co-contaminated with diesel and firefighting agents or dispersant. Researchers of remediation focus on assessing the effectiveness of removal and biodegradation of hydrocarbons in soils but the process of their transport into the atmosphere and water cannot be ignored.
